# A Machine Learning-Based Classification of Immunogenic Cell Death Regulators and Characterisation of Immune Microenvironment in Acute Ischemic Stroke

**DOI:** 10.1155/2023/9930172

**Published:** 2023-11-14

**Authors:** Mengying Wang, Guolian Wei, Shaorui Gu, Zhengyuan Huo, Xue Han

**Affiliations:** ^1^Department of Anesthesiology, Shuguang Hospital Affiliated with Shanghai University of Traditional Chinese Medicine, Shanghai, China; ^2^Department of Neurosurgery, Tongji Hospital, Tongji University School of Medicine, Shanghai 200065, China; ^3^Department of Thoracic Surgery, Shanghai Tongji Hospital, School of Medicine, Tongji University, 389 Xincun Road, Shanghai 200065, China; ^4^Department of Pediatrics, Tongji Hospital, Tongji University School of Medicine, 389 Xincun Road, Shanghai 200065, China

## Abstract

Immunogenic cell death (ICD) regulators exert a crucial part in quite a few in numerous biological processes. This study aimed to determine the function and diagnostic value of ICD regulators in acute ischemic stroke (AIS). 31 significant ICD regulators were identified from the gene expression omnibus (GEO) database in this work (the combination of the GSE16561 dataset and the GSE37587 dataset in the comparison of non-AIS and AIS patients). The random forest model was applied and 15 potential ICD regulators were screened to forecast the probability of AIS. A nomogram, on the basis of 11 latent ICD regulators, was performed. The resolution curve analysis indicated that patients can gain benefits from the nomogram. The consensus clustering approach was applied, and AIS patients were divided into 2 ICD clusters (cluster A and cluster B) based on the identified key ICD regulatory factors. To quantify the ICD pattern, 181 ICD-related dissimilarly expressed genes (DEGs) were selected for further investigation. The expression levels of NFKB1, NFKB2, and PARP1 were greater in gene cluster A than in gene cluster B. In conclusion, ICD regulators exerted a crucial part in the progress of AIS. The investigation made by us on ICD patterns perhaps informs prospective immunotherapeutic methods for AIS.

## 1. Introduction

Data from the Global Burden of Disease (GBD) in 2019 indicate that, after ischemic heart disease, stroke is the second leading cause of tertiary death (11.6% of total deaths) [1]. AIS is the most common form of stroke, and its incidence has been on the rise in recent years [2]. The inflammatory cascade triggered by cell death plays a significant role in the pathogenesis of AIS [3]. The levels of cytokines and oxidative stress markers also drive inflammation, such as IL-1 and IL6 [4–6]. In AIS, hypoxia activates HIF-1*α*, enabling HIF-l*α* the primary pathway to regulate angiogenesis after ischemia [7]. Matrix metalloproteinase-9 (MMP9) and interleukin (IL6, IL8, and IL10) are frequently associated to the prognosis of AIS [8]. Circulating immune cells and brain immune cells play a crucial dual function in the breakdown of the blood-brain barrier following AIS [9].

Immunogenic cell death (ICD) was recognized as a form of regulating cell death (RCD) [10]. This procedure induces an adaptive immune response in the face of necrosis or predetermined death. Numerous in-depth investigations in the clinical utility of ICD have been performed recently. ICD stimulation results in the production of primitive antigenic epitopes and the release of damage-associated molecular patterns (DAMPs) by dying cells [11]. DAMPs can bind to antigen-presenting cells, for instance, dendritic cells (DC), detect and phagocytose dead cell antigens, and deliver them to T cells to excite the adaptive immune response [12]. Synergistic augmentation of immunogenic cell death and macrophage transformation is a novel cancer treatment combo [13]. A novel genetic profile of aneurysms based on ICD features in non-neoplastic illness suggests that ICD patterns and the immune microenvironment are strongly associated with aneurysms [14]. Earlier investigations have merely discovered the value of a modest number of immune cells and immunological-related chemicals in AIS. Nevertheless, AIS's ICD perspective is wanted.

Therefore, we systematically investigated ICD regulators in AIS in this work. Immunoassays between normal tissue and AIS blood samples, as well as a thorough analysis of several subtypes of AIS, will reveal the alterations in the occurrence of ICDs and their associated genes. We developed a gene model to forecast AIS susceptibility on the basis of 11 candidate ICD regulators and observed that patients were capable to get substantial advantages in the model. We made a comparison of biological functions in light of the fact that the two clusters share distinct immunological properties. We found that ICD modification mode exercised a substantial effect on AIS. It would provide brand new information to the investigation into the pathogenic mechanism of AIS.

## 2. Materials and Methods

### 2.1. Data Gathering

The GSE16561 combined GSE37587 dataset of 24 healthy adult subjects and 107 AIS patients were obtained from the GEO database (https://www.ncbi.nlm.nih.gov/geo). Clinical data for both datasets can be found in previous studies [15, 16]. All patients from the two datasets met the following criteria: age ≥18 years, MRI diagnosis of AIS, and blood drawn within 48 hours of onset of stroke symptoms. The datasets were chosen to elucidate gene expression in peripheral whole blood from patients with acute ischemic stroke in order to identify a set of genes for the diagnosis of acute ischemic stroke. Moreover, the data structure and characteristics of both datasets are identical. All patients met the following criteria: age ≥18 years, MRI diagnosis of AIS, and blood drawn within 48 hours of onset of stroke symptoms. Consequently, we combined and normalized the gene expression matrices of the two datasets to investigate the role of ICD-associated genes in acute ischemic stroke. 31 ICD moderators in the terminal normal dataset were annotated [17]: ATG5, BAX, CALR, CASP1, CASP8, CD4, CD8A, CD8B, CXCR3, ENTPD1, FOXP3, HMGB1, HSP90AA1, IFNA1, IFNB1, IFNG, IFNGR1, IL10, IL1B, IL1R1, IL6, LY96, MYD88, NLRP3, NT5E, P2RX7, PDIA3, PIK3CA, PRF1, TLR4, and TNF.

### 2.2. Variations in ICD Regulators among Various Samples and Associated Analysis

We analyzed the variation in the gene expression between normal and AIS samples by applying the “limma” package. We used Spearman's rank association analysis to determine the relationship of the expression of ICD regulators in AIS.

### 2.3. The Establishment of a Random Forest Model and a Nomogram Model

Random forest (RF) and support vector machine (SVM) models were developed by applying the random forest software package as training models to forecast the happening of AIS. “PROC” package performed receiver operating characteristic (ROC) curve as well. We used a ten-fold cross-validation curve to make an estimation of the predictive quality of the RF model for ischemic stroke. The red line stood for the experimental group's error, the black line was on behalf of the error of all samples, and the green line represented the error of the control group. Thereafter, we analyzed the importance of 15 ICD regulators and selected 11 suitable ICD regulators. On the basis of the 11 ICD regulators that were selected, the nomogram model was established by applying the “rms” package. The calibration curve was employed to evaluate the congruence between the anticipated and real values separately. Decision curve analysis (DCA) was carried out, and a clinical effect curve was produced to determine whether the decisions on the basis of the model were beneficial to the patient.

### 2.4. Identification of Molecular Subtypes on the Basis of the Momentous ICD Regulators

Consensus clustering is a kind of technique, which is applied to identify every member and its subgroup figure, as well as to validate clustering rationale on the basis of resampling. Using the “ConsensusClusterPlus” package in R [18], the consensus clustering method was employed to find ICD-related patterns on the basis of the significant ICD regulators.

### 2.5. Identification and Functional Enrichment of Variously Expressed Genes in Various ICD Models

The “limma” package was employed in R and variously expressed genes among diverse ICD patterns were recognized (*p* < 0.01). We employed principal component analysis (PCA) algorithms to calculate out the ICD score for every sample to quantify the ICD patterns. Through applying the “clusterProfiler” package, gene ontology (GO) functional annotation and Kyoto Encyclopedia of Genes and Genomes (KEGG) pathway analysis were made to find out the latent mechanism of variously expressed genes. Gene set enrichment analysis (GSEA) was also performed by the “clusterProfiler” package. The MSigDB C2 set was applied as the reference gene set.

### 2.6. Evaluation of Immune Cell Infiltration

We used the “ESTIMATE” package [19] to measure the immune cell infiltration score in every sample. We made heat maps on account of the gene expression and immune scores of the samples and analyzed the distinctions in immune scores in cluster A and cluster B as well. In addition, we also examined the correlations between significant ICD regulators and immunological ratings.

### 2.7. Statistical Analysis and Visualization

All statistical analyses were carried out by the use of R version 4.1.3. Kruskal–Wallis test was used to make a comparison of the distinctions between groups. The Spearman correlation coefficient was used throughout this study, and significant correlations met the criteria |Spearman *r*| > 0.3 and *p* < 0.05. The “ComplexHeatmap2” and “ggplot2” R packages were employed to visualize.

## 3. Results

### 3.1. Expression Landscape of ICD Regulators among Various Samples

**Figure 1:**
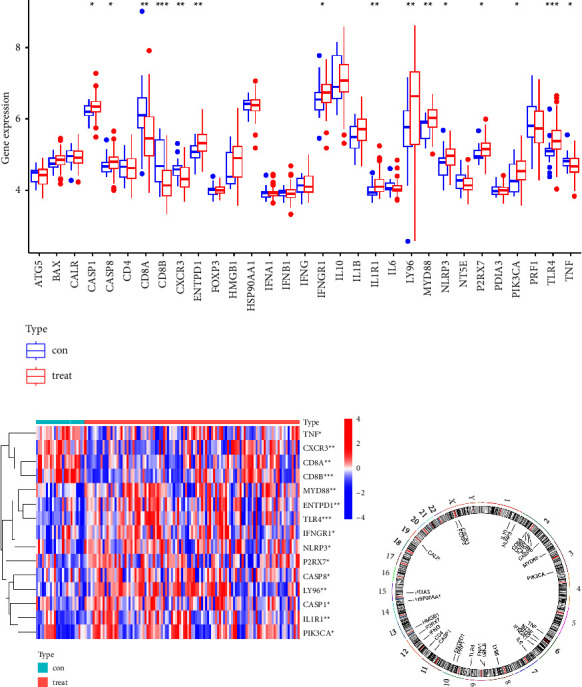
Landscape of the 31 ICD regulators in AIS. (a) Differential expression bar graph of the 31 ICD regulators recognized in non-AIS and AIS patients (^*∗*^*p* < 0.05, ^*∗∗*^*p* < 0.01, and ^*∗∗∗*^*p* < 0.001). (b) 31 ICD regulators' expression heat map in non-AIS and AIS patients. (c) 31 ICD regulators' chromosomal locations.

The participation covered in this survey ranged from thirty-one various ICD regulators.  Figure 1(a) highlighted the AIS samples and the normal samples' expression levels of 15 genes' substantial variations. These genes covered CASP1, CASP8, CD8A, CD8B, CXCR3, ENTPD1, IFNGR1, IL1R1, LY96, MYD88, NLRP3, P2RX7, PIK3CA, TLR4, and TNF. We employed a heat map to depict the distribution of 15 key ICD regulators among the samples that were estimated (Figure 1(b)). It demonstrated each location of 15 regulators in the chromosomes, which was conducted by the “RCircos” package (Figure 1(c)).

### 3.2. Correlation between Different ICD Regulators in AIS

**Figure 2:**
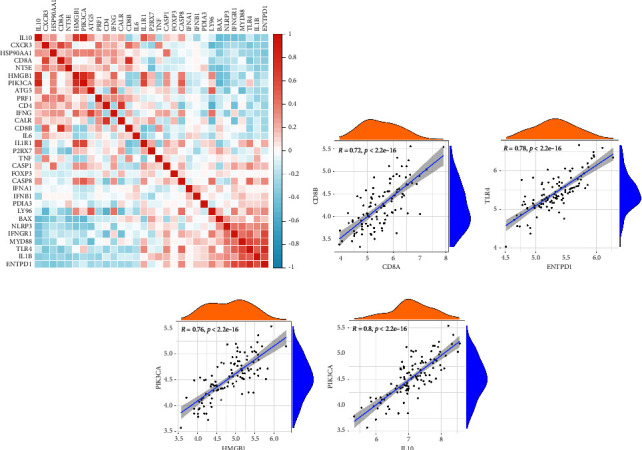
Correlations among 31 ICD regulators. (a) Heat map of association for 31 ICD regulators. (b–e) Four pairs of ICD supervisors with the highest correlation and scatter plots of their correlation (Spearman rank coefficient *r* > 0.7).

In order to study the associate degree that exists among ICD regulators working in various AIS, we analyzed correlation coefficients between ICD regulators based on gene expression and plotted a heat map (Figure 2(a)). Our investigation revealed that IL10, CXCR3, HSP90AA1, CD80A, NT5E, HMGB1, PIK3CA, ATG5, and PRF were related with each other strongly. A strong positive association can also be observed among NLRP3, IFNGR1, MYD88, TLR4, IL1B, and ENTPD1. In addition, substantial positive associations were discovered among four distinct pairs of genes (Figure 2(b)).

### 3.3. Establishment of the RF Model and SVM Model

**Figure 3:**
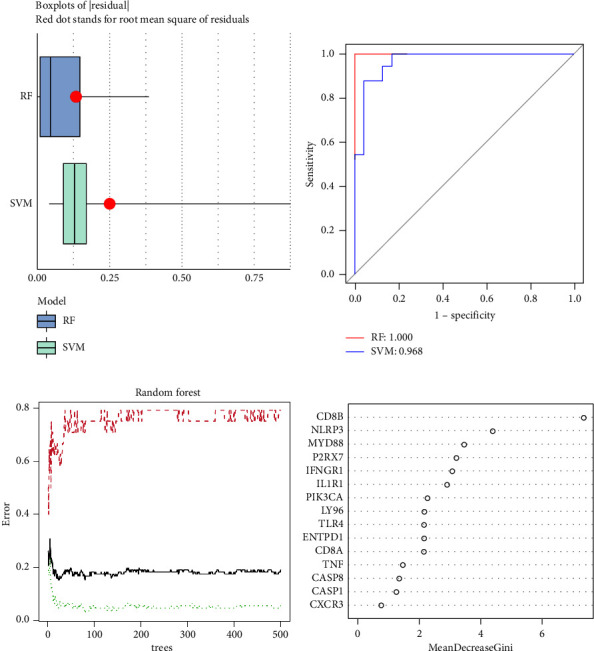
RF model construction. (a) The residual spread of the RF and SVM models was illustrated by employing boxplots of residuals. (b) ROC curves demonstrated the precision of the RF and SVM models. (c) RF model's quality of ischemic stroke forecast was estimated by Ten-fold cross-validation curve. (d) The importance of the 15 ICD regulators on account of RF model.

In order to provide a precise forecast regarding the occurrence of AIS, an RF was established and SVM was selected to choose candidate ICD regulators from the 15 ICD regulators. RF model is a random forest model that generates a final result based on the output of multiple decision trees generated at random. The RF model has a potent capacity for capturing global data characteristics, a strong capacity for model generalization, and the capacity to parallelize calculations swiftly. SVM model is a common classification model that is suited for small samples with clear classification boundaries to determine the optimal segmentation plane. Therefore, these two models are an excellent fit for our investigation. Boxplots of residual, as shown in Figure 3(a), manifested the fact that the RF model's residuals were minimal. The residuals of majority of model cases were correspondingly tiny. Therefore, the RF model was selected as the optimal model to forecast the occurrence of AIS. We established the ROC curve to appraise the model, and its AUC value suggested that the RF model enjoyed more precision than the SVM model (Figure 3(b)). The ten-fold cross-validation curve uncovered a truth that the RF model was most accurate. Nevertheless, we depicted 11 top significant genes of the 15 ICD regulators after these genes were ranked by the means of their significance (Figure 3(d)).

### 3.4. Establishment of the Nomogram Model

**Figure 4:**
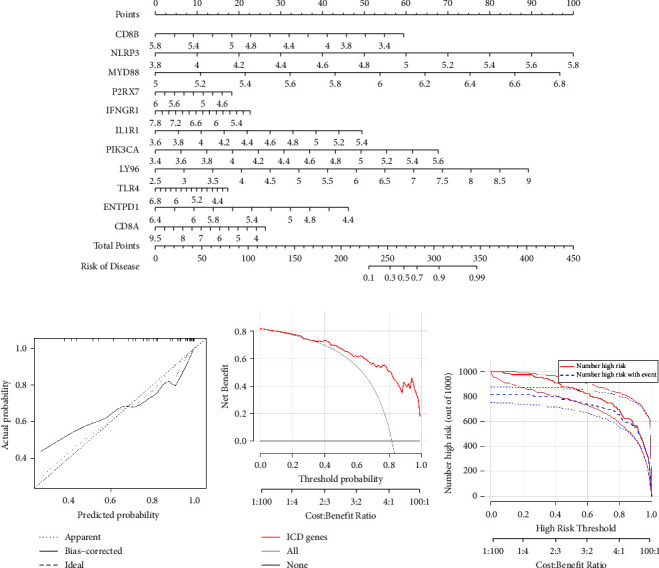
Establishment of the nomogram model. (a) The establishment of the nomogram model on account of the 11 alternative ICD regulators. (b) Calibration curve reveals the predictive capability of the nomogram model. (c) AIS patients may gain advantages from decisions on account of the nomogram. (d) Clinical influence of the nomogram model as estimated by using the clinical impact curve.

On the basis of 11 candidates for the ICD regulations, a nomogram model was created to estimate the prevalence of AIS (Figure 4(a)). CD8B, P2RX7, IFNGR1, TLR4, ENTPD1, and CD8A were protective factors, while NLRP3, MYD88, IL1R1, PIK3CA, and LY96 were hazard elements for AIS. The nomogram model's predictivity was appeared to be accurate by applying calibration curves (Figure 4(b)). The DCA curve demonstrated that judgments, on the basis of the nomogram model, may be beneficial to AIS patients (Figure 4(c)). The clinical influence curve uncovered the nomogram model's outstanding prediction potential capacity (Figure 4(d)).

### 3.5. Significant ICD Regulators Identified Two Distinct ICD Patterns

**Figure 5:**
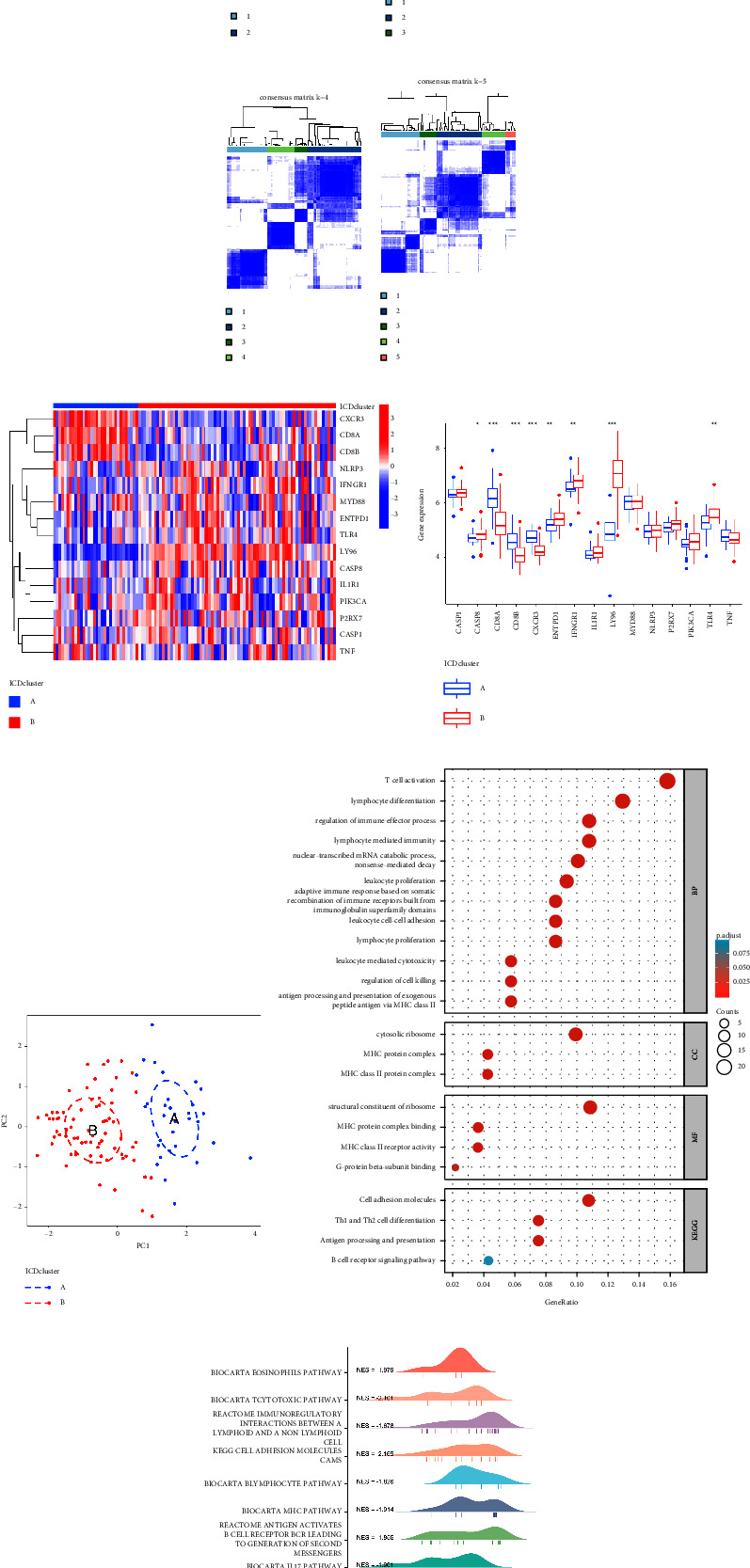
Consensus clustering of the 15 vital ICD regulators in AIS. (a–d) Consensus matrices of the 15 significant ICD regulators for *k* = 2–5. (e) Expression heat map of the 15 crucial ICD regulators in cluster A and cluster B. (f) 15 vital ICD's distinctive expression of regulators in cluster A and cluster B (^*∗*^*p* < 0.05, ^*∗∗*^*p* < 0.01, and ^*∗∗∗*^*p* < 0.001). (g) Principal component analysis recovers transcriptomes' striking distinctions in the two ICD patterns based on the expression profiles of 15 essential ICD regulators. (h) GO and KEGG analyses investigate the possible mechanism based on the influence of the 181 ICD-associated DEGs on the happening and progression of AIS. (i) GSEA analysis examines the latent mechanism based on the effect of ICD-related DEGs on the development and progression of AIS.

The “ConsensusClusterPlus” program was utilized to identify two unique ICD patterns on the basis of 15 major ICD regulators by employing the consensus clustering technique (Figures 5(a)–5(d)). 15 key ICD regulators' expression levels were compared in the two clusters, and afterward a heat map and histogram were generated to indict the variations (Figure 5(e)). There were discernible variations between cluster B and cluster A in the expression level of CD8A, CD8B, CXCR3, ENTPD1, IFNGR1, LY96, and TLR4 (Figures 5(e) and 5(f)). The PCA results revealed that the 15 key ICD regulators were able to differ between the two ICD patterns totally (Figure 5(g)).

A sum of 181 ICD-related DEGs were chosen for prospective investigation between the two ICD patterns. GO functional annotation and KEGG pathway analysis were made for a better comprehension of the latent mechanism behind these DEGs in AIS (Figure 5(h)). We discovered that the majority of the gene sets were enriched in the processes of T cell activation, leukocyte activation, regulation of immune effector, and differentiation of lymphocytes and other processes. We discovered that the differentially expressed genes became enriched in certain biological pathways by using GSEA analysis, for instance, the cytotoxic pathway, the lymphocyte pathway, the cell adhesion pathway, the T cell receptor pathway, the MHC and IL17 pathway, and so on (Figure 5(i)). It suggested that the immunological activity of cluster A was considerably greater than that of cluster B.

**Figure 6:**
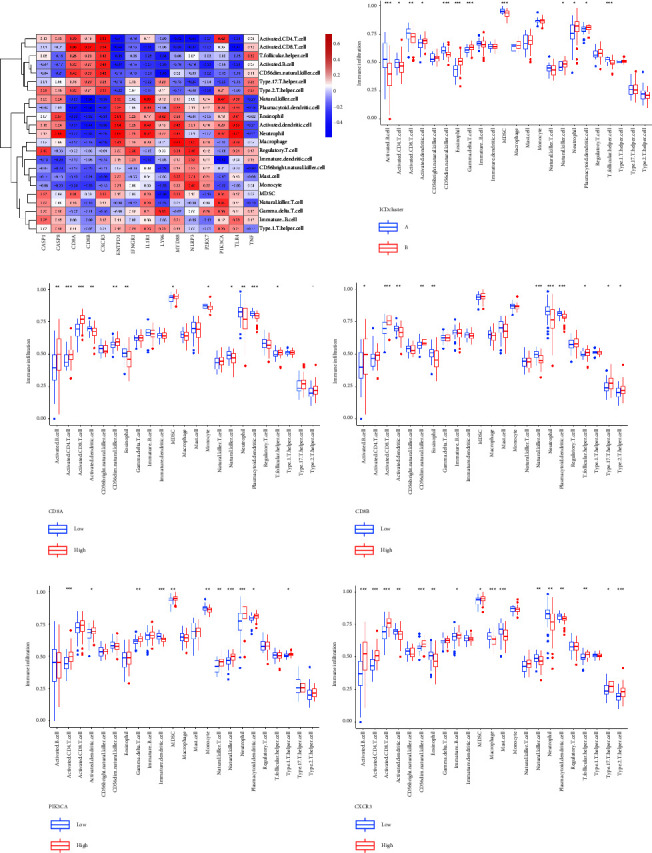
Immune-related enrichment analysis of individual sample gene set (^*∗*^*p* < 0.05, ^*∗∗*^*p* < 0.01, and ^*∗∗∗*^*p* < 0.001). (a) Infiltrating immune cells and the 15 essential ICD regulators' association. (b) Cluster A and cluster B's distinctive immune cell infiltration. (c–f) High and low ICD regulators' distinctive immune cell infiltration, which is evidently linked with infiltrating immune cells.

After that, we utilized “ESTIMATE” to compute the quantity of immune cells presented in the AIS samples, and we investigated the degree to which the 15 most significant ICD regulators which were correlated with the immune cells (Figure 6(a)). We compared the two ICD patterns and observed the distinctions in immune cell infiltration. Neutrophils and eosinophils were more prevalent in cluster B than they were in cluster A, while T cells and MDSC were the opposite (Figure 6(b)). In addition, the link between four major ICD regulators and immune cell infiltration was demonstrated (Figures 6(c)–6(f)). These findings reaffirmed that ICD alteration played a crucial regulatory function in the formation of distinct blood immunological microenvironments in AIS patients.

### 3.6. Recognition of Two Distinct ICD Gene Patterns and Role of ICD Gene Patterns in Distinguishing AIS

**Figure 7:**
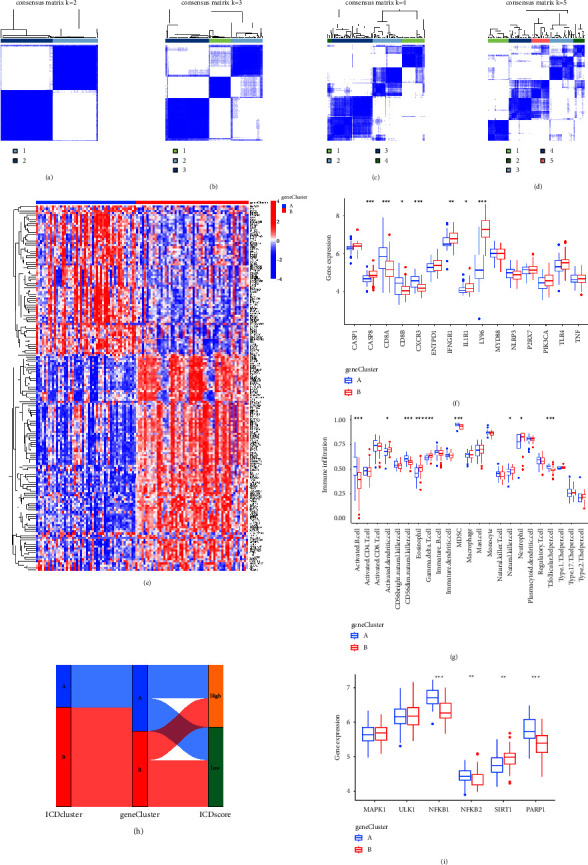
181 ICD-associated DEGs' consensus clustering in AIS (^*∗*^*p* < 0.05, ^*∗∗*^*p* < 0.01, and ^*∗∗∗*^*p* < 0.001). (a–d) 181 ICD-associated DEGs' consensus matrices for *k* = 2–5. (e) Expression heat map of the 181 ICD-related DEGs in gene cluster A and gene cluster B. (f) 15 essential ICD regulators' distinctive expression histogram in gene cluster A and gene cluster B. (g) Differential immune cell infiltration between gene cluster A and gene cluster B. (h) The relationship among ICD patterns, ICD gene patterns, and ICD scores was demonstrated by Sankey diagram. (i) AIS-associated markers' distinctive expression between gene cluster A and gene cluster B.

The consensus clustering method was utilized to classify AIS patients into distinct genomic subgroups on the basis of the 181 ICD-associated DEGs for the prospective validation of the ICD patterns (*p* < 0.05) (Figures 7(a)–7(d)). Two unique ICD gene patterns were identified, which covered gene cluster A and gene cluster B.  Figure 7(e) depicted the expression levels of 181 DEGs that associated to ICD in gene cluster A and gene cluster B. The 15 significant ICD regulators' distinctive expression levels were exhibited as well (Figure 7(f)). Moreover, immune cell infiltration between gene cluster A and gene cluster B was similar to ICD patterns (Figure 7(g)). This proved the precision of the grouping on the basis of the process of consensus clustering once more. We applied a Sankey diagram to describe the association among ICD patterns, ICD gene patterns, and ICD scores (Figures 7(h)). We studied the correlation between ICD patterns and interleukins for the prospective relationship in ICD patterns and AIS. The results illustrated that the expression levels of NFKB1, NFKB2, and PARP1 were higher in gene cluster A than those in gene cluster B, which demonstrated that gene cluster A enjoyed high relevance to AIS (Figures 7(i)).

## 4. Discussion

The transformation of nonimmunogenic cells into immunogenic cells to bring an immune reaction in the course of cell death is referred to as immunogenic cell death (ICD) [20]. ICDs can be triggered by a variety of stimuli, such as viral infections, anthracyclines, certain types of radiation therapy, and photodynamic therapy[21]. DAMPs, generated when cells are stimulated, can attach to pattern recognition receptors (PRRs) on the surface of DC cells, triggering a cascade of physiological events that ultimately activate innate and adaptive immune responses [22]. The ICD pattern was primarily discovered and examined in the field of tumour therapy. Nevertheless, the role that ICD played in AIS was still not fully appreciated.

It is worth noting that inflammation and immunological pathways play a significant role in the pathophysiology of the onset, acute damage cascades, and chronic course of ischemic stroke [23–25]. The mechanism of secondary injury after ischemia may be due to the generation of intracerebral inflammation after ischemic stroke, which accelerates the formation of ischemic injury and affects neuronal mortality and nerve tissue regeneration [26]. Neuroinflammatory response after cerebral ischemia is characterized by activation of microglia, activation of astrocytes, and increase of inflammatory bodies. Malignant edema and hemorrhagic transformation are the most common clinical symptoms, and their mechanisms have been characterized in detail in animal models [27]. A plasma exosome (CircOGDH) has been recognized as a therapeutic target and penumbral biomarker for acute ischemic stroke [28]. Consequently, diagnostic and prognosis evaluation on the basis of peripheral blood biomarkers will exert a significant effect on the mortality control of AIS patients. We believe that our research will contribute to a greater understanding of the crucial role of ICD-related genes and pathways and provide novel diagnosis, prevention, and immunotherapy for stroke patients as well.

First, based on the expression of ICD-related genes, we calculated the ICD score of each sample using the PCA algorithm. Using consensus clustering and the ICD scores of the sample, we then quantified the ICD pattern, resulting in two main ICD clusters. Using the differentially expressed genes between the two ICD clusters, two ICD gene patterns were constructed in order to validate the preceding ICD gene patterns. ICD scores, ICD patterns, and ICD gene patterns are step-by-step analysis results that are used to validate each other. The expression of the majority of the ICD regulators was found to differ significantly between normal and AIS samples in this investigation. We were able to identify ICD regulatory gene patterns on the basis of machine learning models. These gene patterns included CASP1, CASP8, CD8A, CD8B, CXCR3, ENTPD1, IFNGR1, IL1R1, LY96, MYD88, NLRP3, P2RX7, PIK3CA, TLR4, and TNF. AIS samples and normal samples were easily distinguished from one another after the validation of the model, which highlighted the variations in ICD genetic traits between the two types of samples. A nomogram comprised of eleven latent ICD regulators was established, and the DCA curve revealed that the decision on the basis of line graph model could be advantageous to the AIS. CD8B, P2RX7, IFNGR1, TLR4, ENTPD1, and CD8A were protective factors, while NLRP3, MYD88, IL1R1, PIK3CA, and LY96 were hazard elements for AIS.

As an essential component of innate immunity, NLRP3 inflammasome plays a crucial role in the immunological response of the body and the development of illness [29]. It can be triggered by diverse infections or danger signals. A prior work employing bioinformatics and in vivo experiments confirmed that the suppression of IL1R1 or CASP4 ameliorated pyroptosis triggered by NLRP3 inflammasomes [30]. MYD88 performs a critical signal transduction role in innate and adaptive immune responses. Recent research has demonstrated that mesencephalic astrocyte derived neurotrophic factor (MANF) inhibits the production of proinflammatory factors and relies on the TLR4/MyD88/NF-B pathway to maintain the integrity of the blood-brain barrier in a geriatric mouse model following an ischemic stroke [31].

The connection between ICD regulators and AIS immunological features was studied subsequently. The expression of various immune response gene sets and infiltrating immune cells was investigated by the means of using GSEA, ESTIMATE, GO, and KEGG analyses. These immunological characteristics were found to be tightly associated with ICD regulators, which represented that ICD was essential in controlling the blood immune milieu of AIS. Two clusters with distinctive ICD patterns were discovered on the basis of the expression profile of core ICD regulators and ICD-associated DEGs in AIS. Each cluster possesses its unique immunological properties. For example, cluster A patients had a higher proportion of T cells in their blood. The classification of immunological clusters contributes to the elucidation of immune regulation's underlying mechanisms. The association between ICD regulatory patterns and significant immune biomarkers of AIS was also examined. The pathological process of AIS is overwhelmingly tanglesome, including cell excitotoxicity, oxidative stress, cell death processes, and neuroinflammation [32]. Simultaneously, a great number of neurotoxic or neuroprotective signalling pathways are intricately involved in the aforementioned pathophysiological processes. In addition, these signalling pathways have therapeutic potential, as targeting them was likely to be a therapeutic approach for ischemic strokes. The expression features of cytokines regulating inflammatory responses and proteins involved in angiogenesis in the brain and peripheral circulation gave a highlight of the need for the identification of original biomarkers in the circulatory system [33]. Nuclear factor-*κ*B (NF-*κ*B) signalling pathway was essential for maintaining the blood-brain barrier's integrity and therefore was used as a therapeutic target for AIS [34]. As a member of the sirtuin family, SIRT1 regulated a broad physiological process, covering apoptosis and inflammatory reaction, and may be protective factors for stroke [35]. Poly (ADP-ribose) polymerase-1 (PARP-1) regulated cell apoptosis and tissue necrosis in AIS and was associated with prognosis [36]. The findings demonstrated a fact that NFKB1, NFKB2, and PARP1's expression levels were higher, but the expression levels of SIRT1 were lower in gene cluster A than in gene cluster B, which suggested that gene cluster A was strongly correlated with AIS.

This study assessed the function of ICD regulators in patients with AIS. This study demonstrated that ICD regulators can easily differentiate AIS patients from wholesome controls. Two distinct ICD clusters were identified according to 15 ICD regulators, and the model was enhanced by ICD-related DEG expressions, which helped to discovering feasible predictive indicators for the therapy of AIS. ICD expression, immune scores, and biological functional pathways were significantly diverse between the two ICD clusters of AIS. These findings can bring innovative immunotherapeutic concepts for AIS.

Nevertheless, there are certain limitations to the study. First, the data were obtained from a limited sample size GEO data set. It may take a considerable amount of time to collect a great number of samples to get a total comprehension of ICD in AIS. Second, we did not conduct experimental verification because of the difficulties in the process of acquiring AIS samples.

## 5. Conclusion

In conclusion, the study identified 15 potential ICD regulators and a nomogram model, which was capable to forecast the prevalence of AIS with accuracy. This could have significant implications for clinical screening of AIS susceptibility genes and disease course monitoring. Moreover, we discovered significant disparities between the two ICD modes in the blood immune microenvironment. These findings can guide the development of diagnosis and individualized immunotherapies for AIS patients.

## Data Availability

The datasets used to support the findings of this study are available from the corresponding author upon reasonable request.
